# Epigenetic Targets and Their Inhibitors in Thyroid Cancer Treatment

**DOI:** 10.3390/ph16040559

**Published:** 2023-04-07

**Authors:** Ke Zhang, Junyao Wang, Ziyan He, Xian Qiu, Ri Sa, Libo Chen

**Affiliations:** 1 Department of Nuclear Medicine, Shanghai Sixth People’s Hospital Affiliated to Shanghai Jiao Tong University School of Medicine, 600 Yishan Road, Shanghai 200233, China; 2 Department of Nuclear Medicine, The First Hospital of Jilin University, 1 Xinmin St., Changchun 130021, China

**Keywords:** thyroid cancer, epigenetics, target, inhibitor

## Abstract

Although biologically targeted therapies based on key oncogenic mutations have made significant progress in the treatment of locally advanced or metastatic thyroid cancer, the challenges of drug resistance are urging us to explore other potentially effective targets. Herein, epigenetic modifications in thyroid cancer, including DNA methylation, histone modifications, non-coding RNAs, chromatin remodeling and RNA alterations, are reviewed and epigenetic therapeutic agents for the treatment of thyroid cancer, such as DNMT (DNA methyltransferase) inhibitors, HDAC (histone deacetylase) inhibitors, BRD4 (bromodomain-containing protein 4) inhibitors, KDM1A (lysine demethylase 1A) inhibitors and EZH2 (enhancer of zeste homolog 2) inhibitors, are updated. We conclude that epigenetics is promising as a therapeutic target in thyroid cancer and further clinical trials are warranted.

## 1. Introduction

Thyroid cancer is the most prevalent endocrine tumor as well as the ninth most common tumor worldwide, and its incidence has been steadily growing over the past few decades [1]. According to the 2022 WHO Classification of Thyroid Neoplasms, thyroid cancer (follicular cell-derived carcinomas) comprises follicular thyroid carcinoma (FTC), invasive encapsulated follicular variant papillary carcinoma, papillary thyroid carcinoma (PTC), oncocytic carcinoma of the thyroid, high-grade follicular-derived carcinoma and anaplastic follicular cell-derived thyroid carcinoma (ATC) [2]. Most thyroid cancers at an early stage can be cured by conventional therapies such as surgery, ^131^I therapy and TSH suppression therapy, but unfortunately, difficulties in the management of locally advanced or metastatic thyroid cancer still exist, which affect disease-specific survival, mainly due to the fact that thyroid cancer cells are usually resistant to radiation therapy, chemotherapy, multikinase inhibitors and immunocheckpoint inhibitors [3].

Based on the identification of key oncogenic mutations, biologically targeted therapies have undergone rapid evolution in the past two decades, genetic-alteration-specific kinase inhibitors have exhibited favorable efficacy and safety in clinical trials and real-world studies. Nevertheless, primary or secondary drug resistance renders long-term benefits impossible [4]. These challenges have prompted us to probe more deeply into the mechanisms of thyroid carcinogenesis, which is increasingly considered to be influenced by both genetic mutations and epigenetics.

Epigenetics is a set of rules tied to DNA that regulates the interpretation and expression of genes, and epigenetic treatment strategies have shown encouraging early outcomes. For instance, the Food and Drug Administration (FDA) has approved the epigenetic drugs ivosidenib (targeting IDH1) and enasidenib (targeting IDH2) for the treatment of acute myeloid leukemia, both of which lower 2-hydroxyglutarate levels and induce myeloid differentiation, and tazemetostat (an EZH2 inhibitor promoting cell differentiation) for the treatment of follicular lymphoma [5,6]. Considering the good results of epigenetic drugs in other cancers and the reversibility of epigenetic modifications, many scholars looked to epigenetic drugs to deal with the dilemma of thyroid cancer treatment and have made significant headway in recent years.

This review comprehensively discusses the epigenetic modifications and therapeutic targets in thyroid cancer, hoping to provide an explicit update and an outlook for the future in this emerging field.

## 2. Epigenetic Modifications in Thyroid Cancer

**Figure 1:**
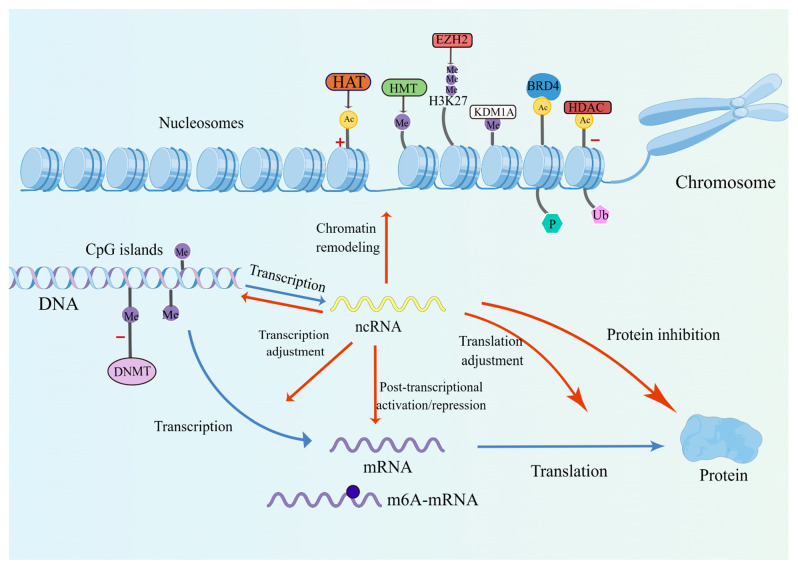
Epigenetic modifications and relevant therapeutic targets in thyroid cancer. DNMT, DNA methyltransferase; Me, methylation; Ac, acetylation; HDAC, histone deacetylase; BRD4, bromodomain-containing protein 4; KDM1A, lysine (K) demethylase 1A; EZH2, enhancer of zeste homolog 2; HAT, histone acetyltransferase; ncRNA, non-coding RNA; P, phosphorylation; Ub, ubiquitination; +, promoting gene expression; −, inhibiting gene expression. Composed with Figdraw.

DNA methylation, histone modifications (including methylation/demethylation, acetylation/deacetylation and bromodomain-containing protein 4 binding acetylated histones) and non-coding RNAs were previously deemed as epigenetic modifications. In recent times, chromatin remodeling and RNA alterations were involved as novel epigenetic modifications. Understanding these epigenetic modifications is helpful to lay a foundation for the exploration of potential targets and the development of therapeutic agents (Figure 1).

### 2.1. DNA Methylation

In eukaryotic genomes, DNA methylation, the addition of a methyl group to the 5-carbon of cytosine, primarily takes place in CpG islands, which are areas with a high number of CpG sites and make up around 70% of human gene promoters. DNA methylation is associated with transcriptional silencing of related genes and is the most common and meaningful DNA modification in thyroid cancer [7]. Genes that regulate cell proliferation and invasion such as *p16INK4A* (cyclin-dependent kinase inhibitor p16), *RASSF1A* (Ras association domain family 1 isoform A), *PTEN* (phosphatase and tensin homolog), *Rap1GAP* (Rap1 GTPase-activating protein), *TIMP3* (tissue inhibitors of metalloproteinase 3), *DAPK* (death-associated protein kinase), *RAR2* (retinoic acid receptor 2) and *E-cadherin* and genes with specific roles in thyroid differentiation such as *NIS* (sodium iodide symporter), *TSHR* (TSH receptor), *pendrin* (sodium-independent chloride/iodide transporter), *SL5A8* (solute carrier family 5 member 8) and *TTF-1* (thyroid transcription factor-1) are typically silenced due to DNA hypermethylation, according to the findings of a number of studies conducted over the years. These processes, which are mediated by DNA methyltransferases (DNMTs), have been shown to play a significant role in the development of thyroid cancer [8]. In addition, a study identified 262 hypermethylated genes in differentiated papillary tumors, 352 in follicular tumors, 86 in anaplastic and 131 in medullary tumors, which demonstrated a difference between thyroid cancer subtypes [9]. However, the underlying mechanisms of those genes and pathways are not completely understood at this time.

### 2.2. Histone Modifications

Histones are the main proteins that make up chromatin and serve as the “spool” around which DNA is arranged [10]. A 147 bp segment of DNA is wrapped around an octamer of histones H2A, H2B, H3 and H4 to form a nucleosome [11]. Post-translational modification of the N-terminal tails of histones includes acetylation, methylation, phosphorylation, ubiquitination, SUMOylation, ADP ribosylation and so on [10]. Among these, histone acetylation and methylation are the most studied histone modifications and have been experimented with corresponding therapeutic targets.

Histone acetyltransferases (HATs) mediate the acetylation of histones, leading to an open chromatin structure and promoting gene expression. Oppositely, histone deacetylases (HDACs) are responsible for deacetylation, leading to a closed chromatin structure and inhibition of gene expression [12]. Both HATs and HDACs carry out their respective functions by affixing themselves to specific lysine residues of histones. HDACs are found in a wide variety of forms; however, they are most commonly organized into the following four classes: class I comprises HDACs 1, 2, 3 and 8; class II comprises HDACs 4, 5, 6, 7, 9 and 10; class III comprises the sirtuins (SIRT1–7); and class IV comprises HDAC 11 [13]. Regrettably, only a limited amount of research has been conducted on the histone changes that are present in thyroid cancer, as well as the relationship between those modifications, and the behavior of thyroid tumors. A recent study, however, has shown that thyroid cancer tissues exhibit altered global levels of histone acetylation [14]. When compared to differentiated tumors, it was discovered that undifferentiated tumors had lower quantities of acetylated H3 at the K18 residue, indicating that acetylation is what turns off the thyroid tumor transformation. In addition to this, a subset of thyroid cancer cells that lost the expression of thyroid transcription factor-1, which is essential for the development of thyroid carcinogenesis, exhibited decreased acetyl-H3-K9 and increased dimethyl-H3-K9 [15]. Incidentally, by attaching to acetylated histones and subsequently influencing gene transcription, the epigenetic regulator bromodomain-containing protein 4 (BRD4) plays an essential role in the onset and progression of many illnesses, including thyroid cancer. Gao et al. assessed the degree of BRD4 expression levels in thyroid tumors and the potential for BRD4 inhibition. In particular, BRD4 was identified to be over-expressed in PTC specimens when compared to normal tissues, pointing to the role of BRD4 in the development of thyroid cancer [16].

Methylation represents another kind of histone modification. Methyltransferases catalyze the addition of methyl groups to proteins, while demethylases catalyze the removal. Lysine and arginine residues in the N-terminal tail of histones are the sites where methylation occurs, leading to mono-, di- or trimethylation of the histone protein [17]. A recent study found that the histone H3 lysine 4 (H3K4) and H3 lysine 9 (H3K9) demethylases (KDM1A) were frequently over-expressed in PTC tissues and cell lines and down-regulation of KDM1A expression inhibited the ability of PTC cells to migrate and invade in vitro and in vivo [18]. It appeared that histone methyltransferases (HMT) such as KMT2D and KMT5A also played a pivotal role in the epigenetic alterations that occur in thyroid cancer [19,20]. Furthermore, it has been established that the polycomb group protein family member enhancer of enhancer of zeste homolog 2 (EZH2), which can result in trimethylation of the histone protein, is specifically up-regulated in ATC cells [21].

### 2.3. Non-Coding RNAs

Non-coding RNAs are divided into two categories based on their length: long non-coding RNAs (more than 200 nucleotides) and small non-coding RNAs (less than 200 nucleotides). Despite the fact that no drugs that target non-coding RNAs are currently available for the treatment of thyroid cancer, non-coding RNAs seem to be promising therapeutic targets that merit additional research in regard to the basic research described below.

Numerous indications point to the possibility that long non-coding RNAs regulate gene expression at several levels, including chromatin remodeling, transcription, genome stability, post-transcriptional alterations and translation. Long non-coding RNAs have a significant role in tumor biology, and their dysregulated expression may contribute to the malignant transformation of cells. A large number of long non-coding RNAs, some of which include NAMA, BANCR and PTCSC3, have been linked to PTC, and these RNAs have the potential to be utilized as biomarkers and potential therapeutic targets in this field [22]. The long non-coding RNA called Prader Willi/Angelman region RNA5 (PAR5) was discovered to be significantly and specifically down-regulated in ATC by Pellecchia and colleagues. Furthermore, the proliferation and migration rates of ATC-derived cell lines were found to decrease when PAR5 was restored. Furthermore, they noted that PAR5 exerted its anti-carcinogenic effect by inhibiting the oncogenic function of the enhancer of EZH2 [23].

MicroRNAs (miRNAs), small nucleolar RNAs (snoRNAs), small nuclear RNAs, piwi-interacting RNAs and small interfering RNAs are all members of the small non-coding RNA family, with miRNAs, which are small molecules consisting of 19–23 nucleotides, being the most well-known and having been widely researched in human malignancies. Endogenous miRNAs have an impact on cell proliferation, differentiation, apoptosis and autophagy, and they have been linked to the onset and development of thyroid cancer through up-regulating or down-regulating the transcription of oncogenes or tumor suppressor genes [24]. For instance, a recent study demonstrated that miR-1246 regulates the PI3K/AKT signaling pathway, which in turn inhibits the proliferation of thyroid cancer cells and the growth of tumors [25]. Moreover, so far, a number of miRNAs have been discovered to regulate radioiodine accumulation and NIS expression in both normal and cancerous thyroid tissues. Among these, miR-146b is one of the most studied miRNAs in thyroid carcinoma. Riesco-Eizaguirre et al. revealed that miR-146b, which is over-expressed in patients with PTC, is beneficial for the conversion from dedifferentiation to differentiation by controlling the miR-146b/PAX-8/NIS circuit for the reinduction of radioiodine accumulation [26]. Additionally, Hou et al. recently showed that miR-146b might modify NIS expression with translocation to the membrane via targeting MUC20 through the MET signaling pathway in dedifferentiated thyroid carcinoma [27]. In addition to this, miR-339, miR-875 and miR-17-92 have also been discovered to enhance radioiodine absorption and NIS expression by modulating the up-regulated miRNA in thyroid cancers [28].

### 2.4. Chromatin Remodeling

Chromatin remodeling involves ATP-dependent repositioning or reconfiguration of the nucleosome, and these changes can alter the dynamic competition between histones and transcription factors for cis-regulatory sequences in gene promoters, with important implications for cell differentiation and tumorigenesis [29,30].

Mutations of subunits of the SWI/SNF (switch/sucrose nonfermentable) chromatin remodeling complexes occur commonly in cancers of different lineages, including advanced thyroid cancers. A recent study discovered that SWI/SNF complexes are central to the maintenance of differentiated function in thyroid cancers, and their loss confers radioiodine refractoriness and resistance to MAPK inhibitor-based redifferentiation therapies [31].

### 2.5. RNA Alterations

Cell differentiation depends heavily on RNA alterations. In eukaryotes, N6-methyladenosin (m6A) is one of the most common modifications of mRNA, controlling a variety of biological activities [32]. m6A modification is mediated by three classes of enzymes: (i) methylators, which include the m6A methyltransferases methyltransferase-like 3 (METTL3), the methyltransferase-like 14 (METTL14) and Wilms tumor 1-associated protein (WTAP); (ii) erasers, comprising the fat mass and obesity-associated protein (FTO) and the alkylation repair homolog protein 5 (ALKBH5); and (iii) readers, which include the YT521-B homology (YTH) domain family (YTHDF1-3 and YTHDC1-2), insulin-like growth factor 2 mRNA-binding proteins (IGF2BP1-IGF2BP3) and eukaryotic initiation factor 3 (EIF3) [33]. Readers recognize m6A, bind the RNA and initiate corresponding functions. These proteins are commonly up- or down-regulated in human cancer tissues to control cell differentiation through changing splicing, RNA processing, protein translation, microRNA binding and RNA–protein interaction [34]. We very recently reported that acquired resistance to differentiation is prompted by IGF2BP2 (insulin-like growth factor 2 mRNA-binding protein 2)-dependent stimulation of ERBB2 signaling. Thus, targeting IGF2BP2 might be a promising strategy to overcome acquired drug resistance in the differentiation therapy of radioiodine-refractory PTC [35]. In addition, we discovered that IGF2BP2 promoted dedifferentiation of PTC by integrating into the 3′-untranslated regions of runt-related transcription factor 2, which bound to the sodium/iodide symporter promoter region and reduced the expression of the protein. Together, these findings pointed to an innovative, differentiated treatment approach that targets IGF2BP2 [36].

## 3. Epigenetic Inhibitors in Thyroid Cancer

For the treatment of thyroid carcinoma, two potential epigenetic approaches have been identified: one involves differentiating tumors to improve their responsiveness to radioiodine therapy, and the other entails de-silencing tumor suppressor genes that can suppress the growth and/or invasiveness of tumor cells [29]. These two mechanisms do not work in opposition to one another, but they do sometimes overlap. HDAC inhibitors, BRD4 inhibitors, KDM1A inhibitors and EZH2 inhibitors have been investigated in vivo (Table 1), while inhibitors of DNMT and HDAC have reached the stage of clinical trials (Table 2).

### 3.1. DNA Methyltransferase Inhibitors

DNA methyltransferase inhibitors, decitabine and azacytidine, are currently only licensed for use in the treatment of myelodysplastic syndromes, not solid tumors [8]. However, preclinical research indicates that restoring the expression of NIS can be accomplished by inhibiting DNA methyltransferases, and this can be accomplished without the necessity of knocking out the mutant form of *BRAF* [49]. In addition, preclinical research conducted in ATC cell lines revealed that decitabine had the potential to increase the expression of MAGEA4 (melanoma antigen family A4), which is a potential target for immunotherapy that is based on T-cell receptors. This finding points to the possibility of a new use for demethylating agents in aggressive PTC patients: altering the immune system [50]. Decitabine is being investigated in a phase II clinical trial (NCT00085293) for the treatment of patients with metastatic PTC or FTC that did not respond to radioiodine. This finding showed that radioiodine uptake in metastatic lesions resumed after treatment with decitabine.

The effectiveness of azacitidine to restore radioiodine uptake in thyroid cancer patients with metastatic or persistent disease was evaluated in a phase I clinical trial (NCT00004062); however, the outcomes have not been made public.

### 3.2. Histone Deacetylase Inhibitors

SAHA, which is also known as vorinostat, is a hydroxamic acid that functions as an inhibitor of HDACs of classes I and II. It was demonstrated to have a direct cytotoxic impact against the BHP7-13 cell line through the process of growth inhibition [51]. When the TPC-1 and BCPAP cell lines were treated with SAHA, the cell viability decreased, *NIS* expression was significantly restored or over-expressed and the oncogene HMGA2 (high-mobility group protein AT hook 2) was negatively regulated [52]. The efficacy of SAHA was evaluated in a phase I study which enrolled six individuals with advanced thyroid carcinoma. After receiving SAHA treatment, one patient had a partial response, while another showed signals of improvement in the radioiodine scan [44]. However, a phase II study did not find any beneficial effects on response in patients with metastatic thyroid tumors that did not respond to conventional therapy [45].

Depsipeptide, which is also known as FK228 or romidepsin, has been used to sensitize anaplastic thyroid cancer cells (SW-1736) to doxorubicin [53]. In addition, a recently published study demonstrated that treatment with depsipeptide on BHP18-21 thyroid cancer cells resulted in differentiation as well as direct cytotoxicity [54]. The National Cancer Institute finished both phase I and phase II of its relevant clinical investigations for thyroid cancer, but the outcomes are still currently unknown [46,47].

Valproic acid (VPA), a short-chain fatty acid, which is already utilized in the treatment of bipolar disorder as well as epilepsy, has been proven to effectively inhibit the catalytic activity of class I HDACs. In a manner analogous to that of SAHA, it was demonstrated to inhibit the proliferation of papillary and follicular cancer cells through the activation of Notch1-related signaling, which ultimately results in the arrest of the cell cycle [55]. In addition, the findings reported by Shen et al. assert that VPA inhibits the proliferation of metastatic follicular cell lines to a significant degree. Unfortunately, it does not trigger apoptosis in ATC cell lines [56]. A phase II clinical research study evaluated the effectiveness of VPA treatment in patients with metastatic, incurable, differentiated thyroid cancer, however, the study failed to demonstrate any objective responses and no increased radioiodine uptake was observed [48].

LBH589, also known as panobinostat, is a hydroxamic acid that potently inhibits the activity of all types of HDAC enzymes. Three ATC cell lines (BHT-101, CAL-62 and 8305C) were in vitro treated with LBH589, which reduced cell viability, prevented colony formation and caused cell cycle arrest and apoptosis. Moreover, in a combined immunodeficiency xenograft model implanted with CAL-62 cells, the cytotoxic effects of LBH589 were verified [37]. Interestingly, a subsequent investigation using these identical animals revealed that LBH589 also prevents cellular invasion and migration by boosting E-cadherin expression and encouraging the E-cadherin/beta-catenin complex’s localization to the membrane [38]. A phase II clinical research study evaluated the effectiveness of LBH589 treatment in patients with metastatic thyroid cancer that are resistant to radioiodine, however, the study failed to demonstrate any objective responses.

Belinostat (PXD101), which has been authorized to treat peripheral T-cell lymphoma, inhibits a wide range of HDACs, including classes I, IIa and IIb. Belinostat was administered intraperitoneally to immunodeficient mice with BHP2-7 xenografts in an in vivo study, and a noticeable tumor inhibition was demonstrated [39].

Trichostatin A is a naturally occurring organic compound that has antifungal and antibacterial properties, as well as the ability to inhibit HDACs of classes I and II. The process of differentiation in BCPAP and TPC1 cells is triggered by trichostatin A, which works by increasing the amount of *NIS* mRNA that is expressed [57]. In addition, Rap1GAP and Pap2, two proteins with a known antiproliferative function, were produced in higher quantities in BCPAP, TPC1 and KTC-1 cells that were treated with trichostatin A [58].

N-Hydroxy-7-(2-naphthylthio)-Hepanomide (HNHA) is a novel anticancer drug that is now being studied in a number of tumors. This drug has been shown to decrease the survival of SNU-790 cells while simultaneously raising a-tubulin and histone H3 acetylation. Through amplification of the pro-form of caspase 3 and enhanced cleavage of pro-caspases 3 and 9, treatment with HNHA causes early apoptosis. In addition, HNHA promotes endoplasmic reticulum stress, which raises intracellular Ca^2+^ levels and arrests the G0/G1 phase. Injecting HNHA into mice with SNU-790 xenografts reduced cellular proliferation and increased survival time without causing systemic toxicity or treatment-related fatalities [40].

In general, despite the fact that preclinical trials demonstrated the proliferation-inhibiting and differentiation-inducing effects of HDAC inhibitors in thyroid cancer, clinical trials with these inhibitors gave unsatisfactory results (Table 2).

### 3.3. Others

BRD4 inhibitors, a recently discovered class of epigenetic modulators, interact with HDACs and control gene expression [12]. JQ1 and I-BET762, two recently identified BRD4 inhibitors, prevented cell cycle arrest in ATC cells by selectively targeting minichromosome maintenance complex 5, suggesting in vivo studies and clinical trials [59]. In particular, JQ1 was evaluated in an ATC mouse model, ThrbPV/PVKrasG12D, and exhibited significant tumor inhibition and improved survival, which are modulated by decreased MYC expression and disrupted cyclin-CDK4/RB/E2F3 signaling [41]. JQ1 was also explored in a PTC mouse model, and it revealed both growth inhibition and restoration of radioiodine uptake, indicating its encouraging applications as an anti-cancer drug [16].

After the exploration of the anti-tumor mechanisms of the lysine demethylase 1A inhibitor in thyroid cancer, Zhang et al. confirmed via in vitro and in vivo study that the highly specific inhibitor GSK-LSD1 considerably slows down the spread of tumor growth and makes it more responsive to chemotherapy. Therefore, they provided a promising treatment strategy for advanced thyroid cancer [42].

Enhancer of zeste homolog 2 inhibitor, EPZ-6438, restored the susceptibility to sorafenib in resistant thyroid carcinoma cells in vitro and in vivo through decreasing the trimethylation of histone H3 at lysine 27 (H3K27me3) and increasing the acetylated lysine 27 of histone H3 (H3K27ac) levels [43]. Therefore, the conclusion can be drawn that the suppression of enhancer of zeste homolog 2 inhibitor represents a potential epigenetic therapy.

These classes of inhibitors above warrant further investigation to explore the therapeutic implications in preclinical and clinical settings.

Other modifications, such as non-coding RNAs, chromatin remodeling and RNA alterations, do not have corresponding inhibitors and are therefore not mentioned here. Moreover, other epigenetic drugs such as enasidenib and ivosidenib are approved by the FDA for the treatment of leukemia, but have not been studied in the treatment of thyroid cancer, which may also provide a direction for future epigenetic therapy research in thyroid cancer [11].

### 3.4. Combined Therapies

Since the results of clinical trials with histone deacetylase inhibitors alone in thyroid cancer were not encouraging, many in vitro studies have explored the efficacy of combined therapies, showing promising results (Table 3). Epigenetic inhibitors of two different mechanisms (e.g., HDACi + DNMTi) or one epigenetic inhibitor in combination with other inhibitors (e.g., HDACi + TKI) are usually explored. Although the underlying mechanisms remain unclear, it seemed that these combinations work better than a single agent, warranting in vivo studies and clinical trials. 

## 4. Conclusions and Future Direction

Among all the epigenetic modifications mentioned above, DNA methylation and histone modifications represent the most potential epigenetic targets for the treatment of thyroid cancer. Although their therapeutic inhibitors remain insufficiently explored by clinical trials, evolving early-stage studies indicate promising perspectives. More research is needed to determine whether epigenetic therapy can contribute to the solution of the thyroid cancer treatment conundrum.

Below are a few potential directions in the field of epigenetic therapy of thyroid cancer: First and foremost, the further exploration of comprehensive epigenetic mechanisms and the link between epigenetics and genetics is of great significance, laying the foundation for epigenetic treatment in thyroid cancer. Second, since the efficacy of single-target drugs is poor due to primary or secondary drug resistance, some epigenetic drugs, such as depsipeptide and EPZ-6438, may present a new option to reduce such resistance. Last but not least, future study of combined therapies is needed based on the promising results of their efficacy in a large number of in vitro trials.

## Figures and Tables

**Table 1:** In vivo studies on epigenetic agents in thyroid carcinoma models.

Drug	Target	Chemical Structure	Model	Type of Cancer	Observed Effect	Ref.
LBH589	HDACs (class I, IIa, IIb, IV)	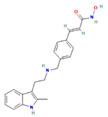	Combined immune-deficiency xenograft model implanted with CAL-62 cells	ATC	Growth inhibition	[37]
			Combined immune-deficiency xenograft model implanted with CAL-62 cells	ATC	Weakening of invasive capacity	[38]
Belinostat	HDACs (class I, IIa and IIb)	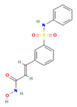	Immunodeficient mice with BHP2-7 xenografts	PTC	Inhibition of tumor	[39]
HNHA	HDAC	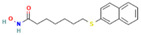	Mice with SNU-790 xenografts	PTC	Proliferation inhibition	[40]
JQ1	BRD4	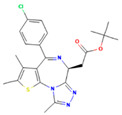	ThrbPV/PVKrasG12D	ATC	Growth inhibition	[41]
			Mice with PTC xenografts	PTC	Growth inhibition and restoration of radioiodine uptake	[16]
GSK-LSD1	KDM1A	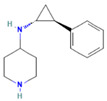	Mice with ATC xenografts	ATC	Growth inhibition	[42]
EPZ-6438	EZH2	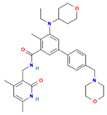	Mice implanted with TC-07 and TC-13 cells	NA	Restoration of sorafenib resistant cells’ sensitivity	[43]

Abbreviations: HDAC, histone deacetylase; BRD4, bromodomain-containing protein 4; KDM1A, lysine (K) demethylase 1A; EZH2, enhancer of zeste homolog 2; PTC, papillary thyroid carcinoma; ATC, anaplastic thyroid carcinoma; TC-07: from the tumor tissues of the sorafenib-sensitive patient; TC-13: from the tumor tissues of the sorafenib-resistant patients; NA, not available.

**Table 2:** Clinical data on epigenetic inhibitors in thyroid cancer treatment.

Inhibitor	Target	Chemical Structure	Phase and Status	Main Result	Ref.
Decitabine	DNMT	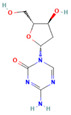	Phase 2, completed	Increase in RAI uptake	NCT00085239
Azacitidine	DNMT	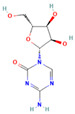	Phase 1, completed	No results posted	NCT00004062
SAHA	HDACs (class I and class II)	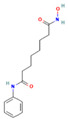	Phase 1, completed	Increase in RAI uptake	[44]
			Phase 2, completed	Faint increase in RAI uptake	[45]
Depsipeptide	HDAC1, HDAC2	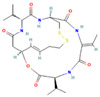	Phase 1, completed	Faint increase in RAI uptake	[46]
			Phase 2, completed	Increase in RAI uptake	[47]
Valproic acid	HDACs (class I)	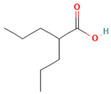	Phase 2, completed	No increase in RAI uptake	[48]
LBH589	HDACs (class I, IIa, IIb, IV)	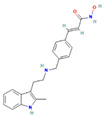	Phase 2, completed	NA	NCT01013597

Abbreviations: DNMT, DNA methyltransferase; HDAC, histone deacetylase; NA, not available.

**Table 3:** In vitro studies on combined therapy in thyroid cancer.

Combined Therapy	Target	Cellular Model	Type of Cancer	Observed Effect	Ref.
SAHA + RDEA119 + temsirolimus + perifosine	HDAC + MEK + mTOR+ Akt	TPC-1, BCPAP, K1	PTC	Growth inhibition and induction of radioiodine uptake	[60]
Depsipeptide + Paclitaxel, or lovastatin, or gefitinib	HDAC + Microtubule + HMG-CoA + EGFR	Primary culture of a papillary thyroid carcinoma harboring BRAF^V600E^	PTC	Growth inhibition	[61]
Trichostatin A + 5-azacytidine	HDAC + DNMT	TPC-1, FTC-133, FTC-236, FTC-238	PTC and FTC	Growth inhibition	[62]
Valproic acid + 5-azacytidine	HDAC + DNMT	TPC-1, FTC-133, FTC-236, FTC-238	PTC and FTC	Growth inhibition	[62]
Valproic acid + TRAIL	HDAC + Death receptor	TPC-1, BCPAP and BHP10-3	PTC	Apoptosis	[63]
Panobinostat + dosatinib or pazopanib	HDAC + MAPK	BCPAP, K1	PTC	Growth inhibition	[64]
Belinostat + Gemigliptin	HDAC + DPP4	BCPAP	PTC	Apoptosis	[65]
HNHA + Levatinib	HDAC + TKI	patient-derived PTC	PTC	Apoptosis, cell cycle arrest and growth inhibition	[66]
Tazemetostat + dabrafenib or selumetinib	EZH2 + MAPK	BCPAP, K1, TPC-1	PTC	Enhancement of differentiation	[67]
PLX51107 + PD0325901	BRD4 + MEK	THJ-11T, THJ-16T	ATC	Apoptosis and proliferation inhibition	[68]

Abbreviations: DNMT, DNA methyltransferase; HDAC, histone deacetylase; MEK, mitogen-activated protein; mTOR, mechanistic target of rapamycin; Akt, protein kinase B; HMG-CoA, 3-Hydroxy-3-MethylGlutaryl-coenzyme A; EGFR, epidermal growth factor receptor; MAPK, mitogen-activated protein kinase; DPP4, dipeptidyl peptidase-4; EZH2, enhancer of zeste homolog 2; TKI, tyrosine kinase inhibitor; BRD4, bromodomain-containing protein 4; PTC, papillary thyroid carcinoma; FTC, follicular thyroid carcinoma; ATC, anaplastic thyroid carcinoma.

## Data Availability

Data is contained within the article.
